# Clinical Applications and Benefits of Using Closed-Incision Negative Pressure Therapy for Incision and Surrounding Soft Tissue Management: A Novel Approach for Comorbid Wounds

**DOI:** 10.7759/cureus.9469

**Published:** 2020-07-30

**Authors:** Treesa W Gomez, Justus W Gomez, Rajesh Gopal

**Affiliations:** 1 Conservative Dentistry and Endodontics, Konaseema Institute of Medical Sciences & Research Foundation, Amalapuram, IND; 2 Electrical Design, WSP Global, Abu Dhabi, ARE; 3 Conservative Dentistry and Endodontics, Rajas Dental College & Hospital, Tirunelveli, IND

**Keywords:** cinpt, negative pressure therapy, wound complications, comorbid wounds, positive pressure infusion, growth factor concentrates, infusion pumps, wound infection

## Abstract

The principle of negative pressure technique dates back to the earliest civilizations; during the Roman era, the technique of using dome-shaped cupping glasses was used to create the suction needed to promote healing. This technique was used throughout the 19th century. In 1821, a British physician named Dr. Francis Fox invented the “glass leech” technique. Thereafter in 1952, an innovative approach was introduced to the treatment of serious, complex wounds through the use of sub-atmospheric or negative pressure known as “negative pressure wound therapy” (NPWT). Later, the “vacuum-assisted closure”, or VAC therapy system founded by Dr. Louis Argenta in 1990 revolutionized the advanced wound care market, and still remains the most clinically proven alternative for the treatment of complex, hard-to-heal wounds. These therapies utilize a foam dressing that is conformed to the wound bed. When sealed and placed under negative (vacuum) pressure, the system creates a unique wound-healing environment that has been shown to promote the wound-healing process, reduce edema, prepare the wound bed for closure, promote the formation of granulation tissue and remove infectious materials. The negative pressure therapy system addresses patient quality of life through an easy-to-use system designed to assist surgeons in the management and treatment of comorbid wounds, and open abdomen and other wound complications to help achieve primary fascial closure. Comorbidities can be defined as a concurrence of multiple chronic diseases in the same patient. Closed-incision negative pressure therapy (CINPT) has revolutionized the way in which caregivers treat the most serious, complex wounds or comorbid wounds. Wound healing can be achieved by the host’s innate and adaptive immune defence mechanisms as in an uninfected simple surgical incision through the skin or by combination of the host’s defence mechanisms and therapeutic modalities. It has been confirmed in some clinical researches that growth factors exert amazing effects on wound-healing promotion and skin function restoration without any obvious side effects. In this review, we have hypothesized a novel modality, focusing on the treatment of wound complications secondary to comorbidity by a combination of negative pressure therapy followed by a positive pressure infusion with growth factor concentrates.

## Introduction

Wound healing is a complex and dynamic physiological process that involves various cells, mediators, extracellular matrix (ECM) components, growth factors, and proteinases [1]. It requires recruitment and differentiation of progenitor/stem cells into tissue-committed somatic cells. Stem cell differentiation is regulated by intrinsic factors and extrinsic micro-environmental cues. On the contrary, infection induces an immuno-inflammatory response and tissue destruction, which hinders the potential of tissue regeneration [2].

Healing of wound consists of three overlapping phases including inflammatory, proliferative, and re-epithelialization phases. Often, the inflammatory phase lasts two to five days after skin damage. Immediately hemostasis is initiated by intravascular platelets, and a clot is formed and bleeding is arrested. Platelets are activated by thrombin and release several growth factors such as epidermal growth factor (EGF), fibroblast growth factor (FGF), transforming growth factors (TGF-α and TGF-β), insulin-like growth factor-1 (IGF-1), platelet-derived growth factor (PDGF), etc. These growth factors diffuse into the wound tissue, serving as biological signals to attract leukocytes, macrophages, neutrophils, and monocytes, which further mediate inflammation. Generally, the proliferative phase takes three days to two weeks after injury, promoting cell proliferation and migration. Proangiogenic factors like PDGF released by platelets and inflammatory cells within the wound area promote new blood vessels and capillaries. The migration of fibroblasts is additionally elicited simultaneously with angiogenesis by the stimulation of PDGF and FGF from inflammatory cells, forming granulation tissue. ECM composed of collagen, proteoglycans, and elastin is produced with the proliferation of fibroblasts. Myofibroblasts play a role in the contraction of the wound area after some of the fibroblasts differentiate into myofibroblasts. Activated keratinocytes around the wound margin migrate to the injured area to complete re-epithelialization. Re-epithelialization varies from three weeks to two years post-injury. Collagen III in newly synthesized ECM is gradually replaced by collagen I and it organizes and augment the tensile strength of healed skin. This phase also involves scar formulation [1].

Complete regeneration without a scar, following injury in humans, can occur only in the pre-natal foetus within 24 weeks of gestation. Post-natal wounds always heal by repair or by a combination of repair and regeneration. Some lower vertebrates, such as the salamander and zebrafish, have a remarkable capacity to regenerate entire limbs, the lens of eyes, and portions of the heart [2].

## Technical report

Negative pressure wound therapy

Masden et al. studied that there is a significant rate of postoperative infection and dehiscence in patients with multiple comorbidities [3]. Surgical site infection and other common surgical site complications (dehiscence, hematoma, and seroma formation) can cause serious and often life-threatening complications. Gauze, adhesive dressings, and skin adhesives have traditionally been utilized for incision management. However, negative pressure wound therapy (NPWT) over clean, closed surgical incisions (closed-incision negative pressure therapy [CINPT]) has become a recent option for incision management [4].

NPWT features a positive effect on open and complicated wounds. A growing body of the literature has reported the benefits of NPWT over closed incisions to help reduce complications in high-risk groups. Therefore, it has been used for “at-risk surgical incisions” with the aim of redistributing lateral tension and holding incision edges together. There is little comparative evidence for the use of CINPT in acute incisions of general or colorectal surgical patients considered to be at high risk of wound complications secondary to comorbidity. CINPT may diminish the risk of external wound contamination, since the dressing is sealed and applied in a sterile environment [5]. The prevalence of people suffering from chronic wounds has risen sharply in recent years, due to the dramatically increasing incidence of obesity and chronic diseases such as diabetes and venous and arterial insufficiency. Chronic wounds affect about 2% of European and US populations; for example, the prevalence of diabetic ulcers alone has already reached as high as 10%-22% in diabetic patients. However, traditional therapies generally involve costly and long-lasting treatments with a high ulcer relapse rate of above 70% [1].

NPWT has mostly been used to manage acute or chronic open wounds through such mechanisms as protection of the wound bed, splinting of soft tissues, reduction of edema, increased perfusion, and enhanced formation of granulation tissue [5]. Vacuum-assisted closure (VAC) therapy utilizes an open-cell polymer foam dressing that is conformed to the wound bed. There is consensus that intact skin should not be exposed to polyurethane foam because the foam can excoriate and blister the tissue [6]. A nonadherent layer is recommended between the foam and the incision [5]. Typically, CINPT dressings do not need to be changed throughout the seven-day duration of therapy. The decreased frequency of dressing change is particularly advantageous in obese patients or in patients with difficult-to-access incisions [5]. Numerous experimental studies are being conducted to study the cause-effect relationships between the mechanical signals and the transduction pathways that result in an improved granulation response. A three-dimensional finite element model was developed by Wilkes et al. to accurately quantify the tissue microdeformations during therapy. This was used to study the effect of the dressing type and subatmospheric pressure level on variations in the microdeformational strain fields in an artificial dermal wound bed [7].

The ultimate goal of wound management is to prevent serious infection, accelerate wound healing, and reduce scars and pain for patients. Repair and regeneration is regulated by cell-to-cell and cell-to-extracellular matrix cross-talk and by the expression of growth factors/cytokines and other bioactive molecules at different temporal and spatial stages during wound healing [2]. Topical therapeutic agents contain growth factors and antimicrobial agents, being crucial for the wound treatment and skin regeneration.

Positive pressure infusion

Comorbid wounds are usually deficient in many of the required adjuvants for physiological wound healing. Therefore, these adjuvants need to be transported to the wound site. One of the ways to provide them is through infusion.

An infusion system may be a device, and any associated disposables, delivering fluids or drugs in solution to the patient. A fluid reservoir maintains the infusate for delivery by a positive pressure displacement pump. Infusate is drawn by the pump into a drug pressurant chamber. A check valve prevents backflow into the reservoir [8]. In general, infusion devices can be divided into two main groups: the gravity flow infusion devices and infusion pumps. A gravity flow infusion device relies on the gravitational force exerted by a liquid column to push the fluid via a venous access into the patient’s bloodstream, whereas an infusion pump has a motorized pumping mechanism to generate the positive pressure. Within the gravitation group are the manual gravity flow sets and the infusion controllers [9].

There are several types of disposable infusion pumps, including elastomeric, positive pressure (spring powered and gas pressure powered), negative pressure (vacuum), etc.

Elastomeric Infusion Pumps

In all elastomeric disposable devices, the pressure on the fluid is generated by the force of a stretched elastomer.

Spring-Powered Infusion Pumps/Positive Pressure Spring-Powered Pumps

These are powered by the energy stored in a compressed spring, the flow rate being significantly higher at the beginning of infusion than at the end. These variations are due to fluctuations in the pressure applied on the fluid by the compressed spring; the pressure decreases with decreases in the volume of the drug reservoir.

Negative Pressure Infusion Pumps

With negative pressure pumps, a driving force is generated from the pressure difference across two sides of the pump's low-pressure chamber wall, with one side being at very low pressure (inside a vacuum chamber) and another being at atmospheric pressure [8].

Infusion Pump System With Controls

Duran et al. introduced an infusion pump system in which the serum amount is controlled and delivery of the required amount of serum to the patient is ensured. The system is designed as to close itself when the serum flow amount reaches the predetermined limit value, and designed as to give an audible warning when a problem occurs within this process [9].

The accuracy of each pump's flow depends on several factors, including temperature, fluid viscosity, atmospheric pressure, back pressure, partial filling, and storage. With reference to the atmospheric pressure, although the driving mechanisms of spring and balloon pumps are not directly dependent on atmospheric pressure for their operation, their flow accuracy can be significantly affected by changes in ambient pressure. Negative pressure devices are predictably affected by variations in the atmospheric pressure. Disposable infusion pumps can be used in many areas, including home care, patient-controlled analgesia, patient-controlled epidural analgesia, continuous peripheral analgesia, continuous epidural analgesia, continuous IV analgesia and pediatric applications. The pressure generated by disposable pumps on fluid is 250-600 torr, compared with 5-1200 torr of pressure for electric pumps [8].

Positive pressure infusion with growth factor concentrate and updates in wound management

The population with comorbidities differs from that with individual chronic diseases. The interaction between multiple conditions results in the necessity for a comprehensive and multidisciplinary approach and consistent continuing care [10]. Growth factors are biologically active polypeptides that regulate cell growth, differentiation, and migration and exert an impact on all stages of wound healing. Some clinical researches confirm that growth factors exert amazing effects on wound healing promotion and skin function restoration free from obvious side effects [1]. For the stimulation of tissue repair, platelet-derived fractions have been used as an autologous source of growth factors and biomolecules, namely, platelet rich plasma (PRP), platelet poor plasma (PPP), and platelet rich fibrin (PRF). The continuous release of growth factors from these concentrates has been proposed to promote angiogenesis both in vitro and in vivo [11]. Previously, PRP was used to seal incisions. In plastic surgery PRP has emerged as an effective treatment adjunct for cutaneous wounds and fat grafts. An eye-shaped PRF clot has been used for the surgical repair of corneal perforation.

The standard surgical threads could be bio-activated with genetically modified microalgae to release both recombinant growth factors and oxygen directly into the wound site. Challenges of using exogenous growth factors is that the levels of matrix metalloproteinases are upregulated that hinder wound healing by degrading growth factors [12]. Therefore autologous growth factors concentrates such as PRP, PRF, etc. may be preferred to exogenous growth factors.

PRP demonstrated higher blood perfusion in the lesion as well as a more mature granulation tissue when compared to those treated with PPP. Injection of these products within the injured muscle tissue of mice induced the reperfusion of blood into the lesion [11]. Therapeutic cerebral angiogenesis, utilizing angiogenic factors to reinforce collateral vessel formation within the central nervous system (CNS), may be a potential method for cerebral revascularization. A previous dose-response study determined that the intracerebroventricular infusion of vascular endothelial growth factor (VEGF) increases vascular density with minimal associated brain edema at a concentration of 5 μg/ml [13]. It was found that titanium-prepared PRP (TPRP) has better angiogenic potential than its counterpart PRP [14]. These novel growth factor concentrates may be further studied for their effects on wound healing and their administration may be incorporated in CINPT.

Novel hypothesis

Since normal physiological wound healing is difficult to achieve in comorbid wounds due to the discrepancy in necessary molecules, a supporting infusion system needs to be introduced.

**Figure 1:**
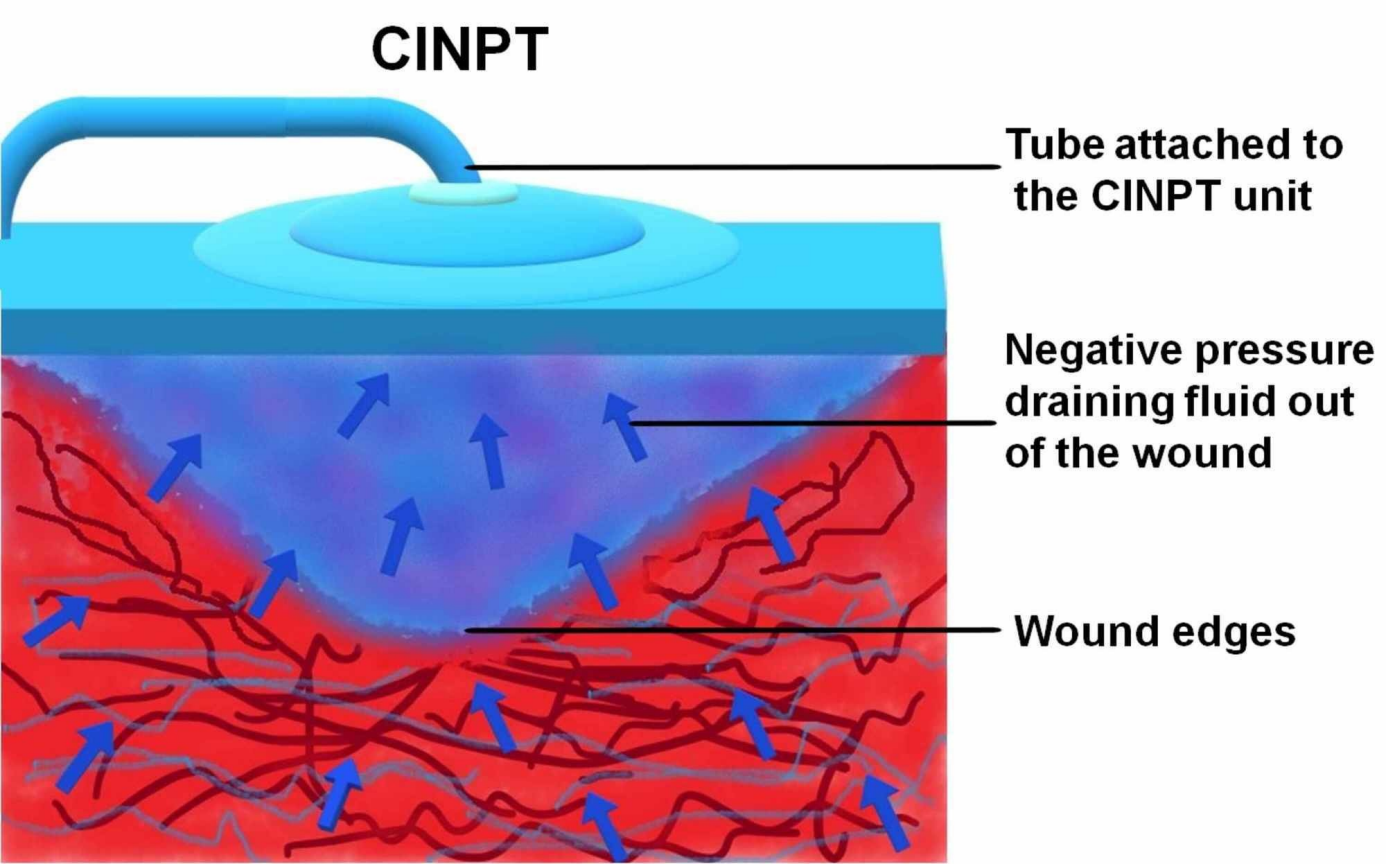
Closed-incision negative pressure therapy (CINPT) Arrows represent the drainage being carried out of the wound, through the tube attached to the foam dressing.

**Figure 2:**
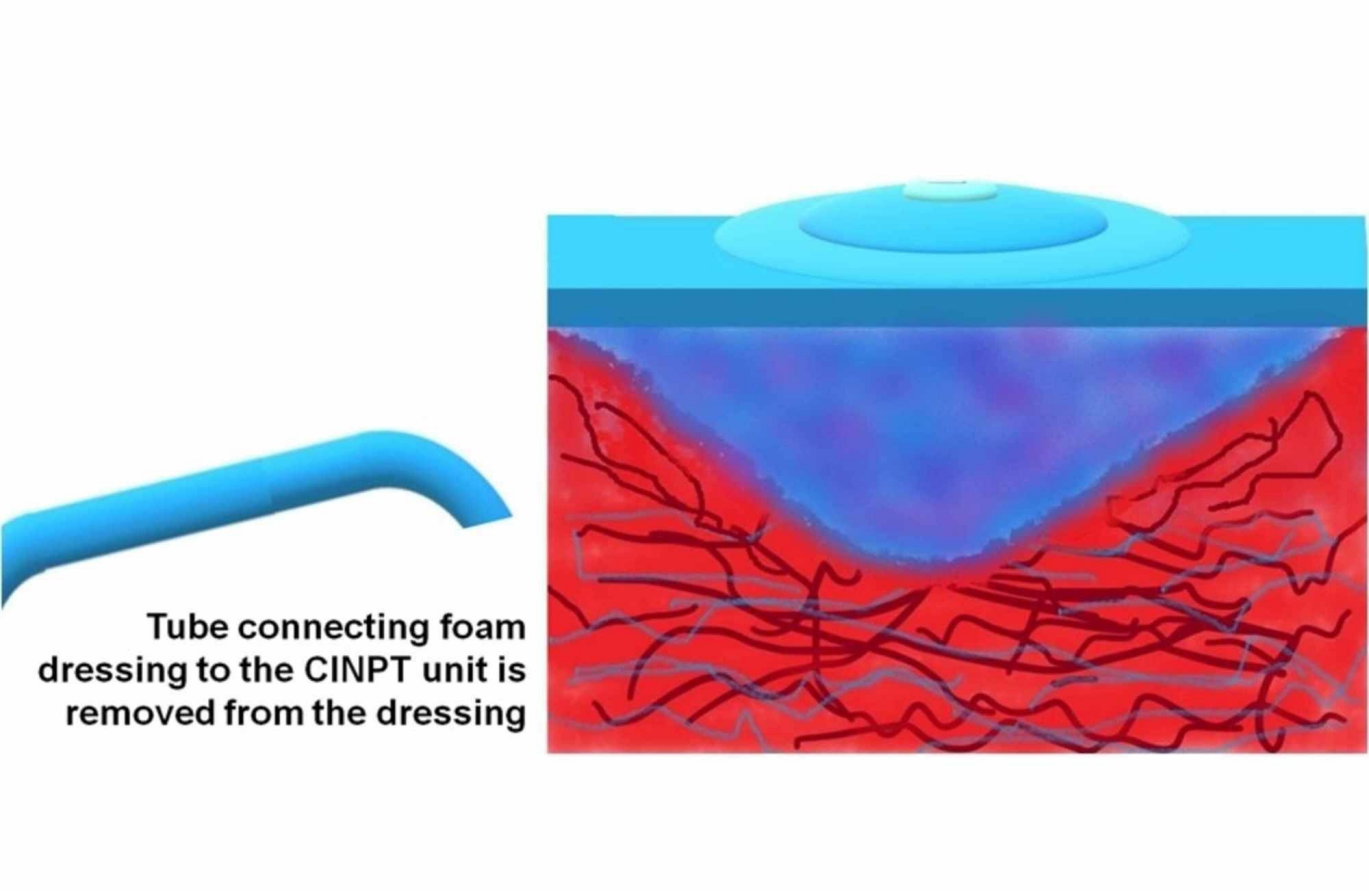
Disconnection of “tube to the CINPT unit” CINPT, closed-incision negative pressure therapy.

**Figure 3:**
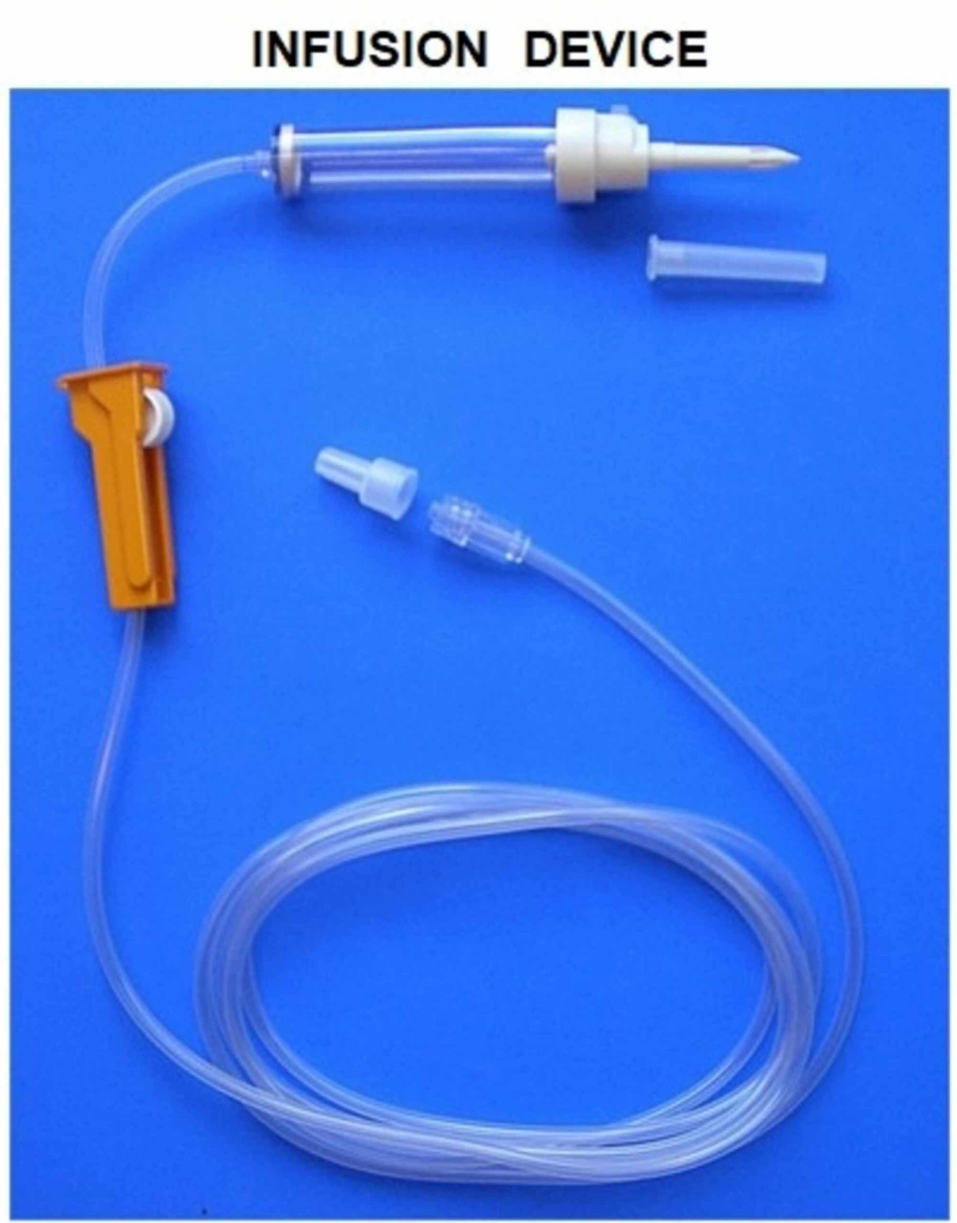
Infusion device showing the infusion tube that can be attached to a pump to regulate the delivery of growth factors

**Figure 4:**
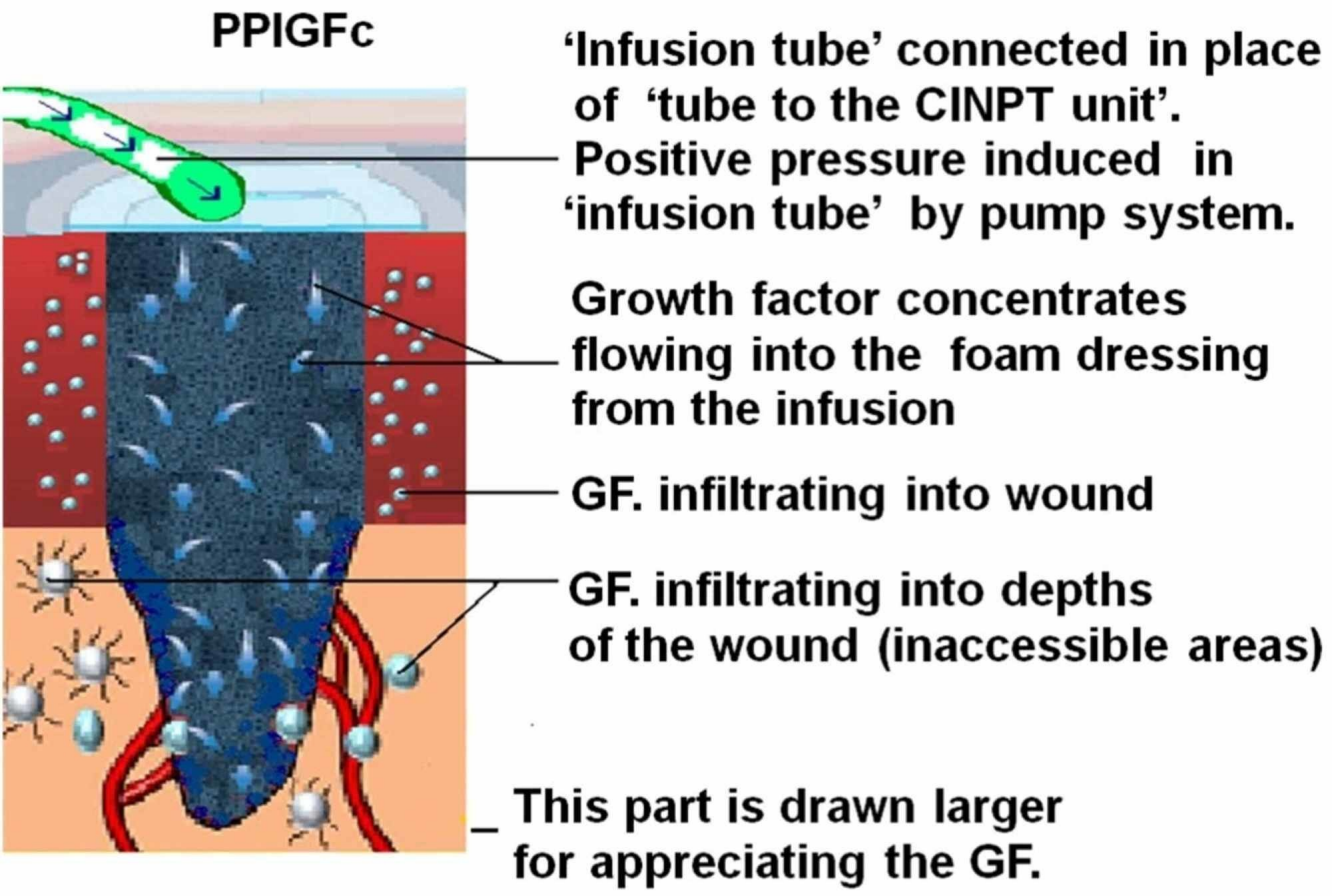
Positive pressure infusion with growth factor concentrate (PPIGFc) GF, growth factor. Here, the GF concentrate is shown infiltrating into the wound depths.

CINPT dressings do not need to be changed throughout the seven-day duration of therapy, and reports suggest that the decreased frequency of dressing change is especially advantageous in obese patients. Since the proliferative phase of wound healing generally takes three days to two weeks after incision, featured with cell proliferation and migration [1], a positive pressure infusion with growth factor concentrate (PPIGFc) will be required in this phase of wound healing. Figures 1-4 explain “wound management for complications secondary to comorbidity” through a simple method by which CINPT can be followed by PPIGFc. First, the wound is subjected to CINPT (Figure 1). After a period of CINPT, this tube is disengaged from the foam (Figure 2). This tube is then substituted with a positive pressure infusion tube and connected to the foam dressing (Figure 3). An infusion of autologous growth factor concentrates is introduced through the infusion tube to the wound site (Figure 4). A positive pressure pump can regulate the inflow of growth factor concentrates depending upon the requirement of each individual patient. Therefore, we can conclude that three to seven days of CINPT can be followed by one to two weeks of PPIGFc for wound complications secondary to comorbidity. This permits a continuous flow of preferably “autologous” growth factors through the foam dressing, into the wound depths and inaccessible areas. By PPIGFc, the stable cells can be stimulated into the labile cells and enter the cell growth cycle, thus aiding in faster healing.

 

 

## Discussion

Few adjuvants in wound healing

The topical application of EGF promotes cell proliferation, differentiation, and migration and accelerates epidermal regeneration. The topical application of PDGF increases the structural integrity of vessels and promotes cell proliferation, ECM deposition and re-epithelialization. The topical/subcutaneous injection of granulocyte-macrophage colony-stimulating factor (GM-CSF) promotes the local recruitment of inflammatory cells, and stimulates cell proliferation and differentiation and wound contraction. The topical application of basic fibroblast growth factor (bFGF) promotes collagenase production, ECM deposition and re-epithelialization. The topical application of TGF-β promotes granulation tissue formation, re-epithelialization, matrix formation, and remodeling [1].

The topical administration of growth factors after debridement is a favourable approach to strengthen wound healing due to the cellular deficiency or a noticeable deterioration of quality in chronic wounds. PDGF is the first and only recombinant growth factor approved by the FDA in USA for topical administration and is used for the treatment of diabetic foot ulcers (but overdosages are reported to have an increased risk of cancer). VEGF-A improves re-epithelization of diabetic foot wounds associated with enhanced vessel formation. EGF augments healing of skin grafts following partial-thickness burns. Studies conducted on mice suggested TGF-α in early re-epithelization. FGF-10 has been successful in improving the healing rate of non-healing venous ulcers [12].

The use of hydrosol/scaffold like titanium dioxide (TiO_2_) strongly inhibits the growth of *Staphylococcus aureus* and induces red blood cell aggregation to stop bleeding. According to a report by Fan et al., a nanofibrous scaffold carrying nano-TiO_2_ hydrosol was designed for better skin repair [15]. Due to the embedment of nano-TiO_2_, the scaffold strongly inhibited the growth of *S. aureus* and induced red blood cell aggregation to stop bleeding. Collagen-chitosan (COL-CS) porous scaffolds have been widely used as a dermal analogue to induce fibroblast infiltration and dermal regeneration. To improve anti-bacterial properties, nano-TiO_2_ hydrosol was introduced into COL-CS scaffolds. TiO_2_/COL-CS porous scaffolds were fabricated through a freeze-drying process, and scanning microscopy (SEM) was employed to observe the micro-structure of the scaffolds. Fourier transform infrared spectroscopy (FT-IR) determined the intermolecular interactions within the scaffolds. The results showed that the scaffold is comparatively more permeable and it may provide a moist environment for wound repairing. The degradation in the lysozyme solution for four weeks showed that porous scaffolds are stable, which can satisfy the wound coverage protection within the repair period [15].

Injectable nano-engineered hydrogels enhance cell adhesion and spreading, increase platelet binding, reduce blood clotting time, and facilitate in vitro tissue regeneration and wound healing. Chitosan and poly(ethylene oxide) are electrospun into nanofibrous meshes as mimics of ECM. Nanofiber/nanoparticle scaffolds significantly accelerate tissue regeneration and remodeling and promote angiogenesis [1]. These adjuvants may be able to hasten wound healing when used in combination with CINPT, but the therapeutic protocols are yet to be studied.

Effects of pressure differences on wound healing

Liu et al. compared effects of negative pressure and positive pressure in wound healing by creating a homemade device. They found that negative pressure and positive pressure promote an inflammatory response and upregulated the expression of growth factors such as EGF, VEGF, PDGF, TGF-β1. Both negative pressure and positive pressure significantly increased cell proliferation in the wound tissue on day 10 compared to the controls. They measured the number of endothelial cells in wound tissue by platelet endothelial cell adhesion molecule 1-CD31-positive staining. Positive pressure increased the number of CD31-positive cells more significantly than the negative pressure in the wound tissue from days 3 to 10. A lower amount of mesenchymal stem cells in the wound tissue was detected following exposure to negative pressure in wound healing when compared to positive pressure therapy [16].

In vitro studies have revealed that cells allowed to stretch tend to divide and proliferate within the presence of soluble mitogens, whereas retracted cells remain quiescent. It had been hypothesized that the application of micromechanical forces to wounds can promote wound healing through cell shape-dependent, mechanical control mechanism. A simulated VAC application was developed to study the effects of vacuum-induced material deformation and it was concluded that the tissue deformation stretches individual cells thereby promoting proliferation in the wound microenvironment. Application of micromechanical forces could also be a useful method to stimulate wound healing through the promotion of cellular division, angiogenesis and native elaboration of growth factors [17].

Advances of positive pressure infusion/nano-drug delivery system

Gillies et al. developed a positive pressure infusion for the clinical means of achieving convection-enhanced delivery of therapeutic agents within the tissues of the CNS for the treatment of glioblastoma multiforme and other diseases of the brain [18]. Despite enormous advances in drug therapy, the use of nanotechnology-based systems has revolutionized the field of drug delivery, offering the possibility to deliver therapeutic agents to local areas in the brain [19].

Chronic wounds remain a challenge because current therapies mostly fail to provide favorable outcomes in wound healing. The nano-drug delivery systems (DDSs) has brought a new insight into skin regeneration of wounds. These drug carriers prolong drug release, protect drug from degradation, and improve skin retention. They have immense potential in the enhancement of drug therapeutic efficacy for their potential in preventing drug degradation and sustaining drug release. Nano-DDSs carrying therapeutic agents are being studied and manufactured, mainly including liposomes, polymeric nanoparticles, inorganic nanoparticles, lipid nanoparticles, nanofibrous structures, and nanohydrogel [1].

Few molecular events in wound healing

Chronic medical conditions are significantly more prevalent among older adults [10]. The Hayflick limit is the amount of times that a normal cell population divides before it stops dividing. Each time a cell divides, the length of telomeres is shortened owing to the loss of pieces of telomeres. When the length of telomeres becomes shortened to a critical point, the cell is prevented from dividing. This is called replicative senescence [2]. In the cell growth cycle, stable cells are neither cycling nor dying. They can be induced to re-enter the cycle by an appropriate stimulus. Although growth can be accomplished by shortening the cell cycle, etc., the most important factors are those that recruit stable cells into the cell cycle. Polypeptide growth factors present in the serum or produced by cells are the most important factors. Cell growth is initiated by the binding of a putative growth factor to specific receptors either in the cell or outside the cell. Most growth factor receptors are equipped with intrinsic protein tyrosine kinase activities that are activated after ligand binding. Such receptors have a large glycosulated extra-cellular ligand binding domain, a single hydrophobic transmembrane region, and a cytoplasmic domain that contains the tyrosine kinase activity. Ligand binding induces a conformational alteration of the extra-cellular domain, which in turn induces dimerization of receptors. The net result is the activation of a protein phosphorylation cascade that stimulates the stable cells to re-enter the cell growth cycle [20].

## Conclusions

Certain comorbid conditions are known to potentially increase the risk of surgical wound complications. Comorbid patients usually have a deficient immune system that leads to a deficient repair system. In such patients, a combination of CINPT and positive pressure infusion with autologous growth factor concentrates (PPIGFc) is hypothetically advocated. This equips the tissues with growth factors thereby compensating their deficiency. The procedure can be easily done by disengaging the negative pressure tube and substituting it with the tube of a positive pressure infusion of growth factor concentrates. Therefore, it is not necessary that the dressing needs to be changed and the rate of infusion can be regulated by any of the types of pumps as is required by the condition of the patient. The protocol can be CINPT followed by PPIGFc, vacuuming away the byproducts of inflammation and inhibiting bacterial growth and then bathing the wound area with a continuous supply of growth factors thereby stimulating the stable cells to begin regeneration. Although further researches need to be implicated, a combination of CINPT, for aiding in wound closure, and PPIGFc, for revascularisation of the surgical site, may aid in the better management of postoperative infection and dehiscence in patients with multiple comorbidities.
